# Patients' knowledge and concerns about using the implantable cardioverter defibrillator for the primary prevention of sudden cardiac death and its correlates: A cross‐sectional study

**DOI:** 10.1002/hsr2.698

**Published:** 2022-06-13

**Authors:** Mohammad A. Zakeri, Nadia Sedri, Golamreza Bazmandegan, Maryam Zakeri, Mohammad Safariyan, Mahlagha Dehghan

**Affiliations:** ^1^ Social Determinants of Health Research Centre Rafsanjan University of Medical Sciences Rafsanjan Iran; ^2^ Non‐Communicable Diseases Research Center Rafsanjan University of Medical Sciences Rafsanjan Iran; ^3^ Critical Care Nursing, Zarand Nursing Faculty Kerman University of Medical Sciences Kerman Iran; ^4^ Clinical Research Development Unit, Ali‐Ibn Abi‐Talib Hospital Rafsanjan University of Medical Sciences Rafsanjan Iran; ^5^ Department of Family Medicine, Ali‐Ibn Abi‐Talib Hospital, School of Medicine Rafsanjan University of Medical Sciences Rafsanjan Iran; ^6^ Physiology‐Pharmacology Research Center, Research Institute of Basic Medical Sciences Rafsanjan University of Medical Sciences Rafsanjan Iran; ^7^ Department of Cardiology, Faculty of Medicine Rafsanjan University of Medical Sciences Rafsanjan Iran; ^8^ Department of Critical Care Nursing, Razi Faculty of Nursing and Midwifery Kerman University of Medical Sciences Kerman Iran

**Keywords:** barriers, heart failure, quality of life, self‐efficacy, social support, sudden cardiac death

## Abstract

**Background and Aims:**

Sudden cardiac death (SCD) is one of the most common causes of mortality in heart failure (HF) patients with reduced ejection fraction. Patients have concerns about the disease and use the implantable cardioverter defibrillator (ICD) to reduce the effects of HF disease. The current study aims to evaluate the barriers and factors affecting the implantation of the ICD for primary prevention.

**Methods:**

One hundred‐forty‐seven patients with HF were studied in public hospitals in southern Iran by using a cross‐sectional design from April 2018 to June 2019. Demographic, researcher‐made questionnaire, World Health Organization Quality of life‐BREF (WHOQOL‐BREF), general self‐efficacy questionnaires, and Multidimensional Scale of Perceived Social Support (MSPSS) were measured for investigating the barriers and impact factors in patent HF.

**Results:**

Most participants were male (56.5%), married (88.4%), illiterate (54.1%), and unemployed (72.6%). 62.6% (*n* = 92) of the participants did not know about HF and ICD. The total score of patients' concerns about using ICD was 47.11 ± 11.26, which showed a moderate level. The scores of knowledge about HF and ICD had a significant positive poor correlation with self‐efficacy, perceived social support and QoL. Also, the score of concerns about the ICD had a significant negative poor correlation with perceived social support.

**Conclusion:**

Understanding HF patients' issues and obstacles can help us prevent sudden death. Doctors' advice has a significant impact on patients' acceptance. Poor knowledge is the most important reason for nonparticipation. Intervention is necessary to inform patients to understand the advantages and disadvantages.

**Abbreviations**
ACC: American College of Cardiology
AHA: American Heart Association
CCU: critical care unit
CIED: cardiovascular implantable electronic device
HF: heart failure
HRS: heart rhythm society
ICD: implantable cardioverter defibrillator
MRI: magnetic resonance imaging
SCD: sudden cardiac death

## 1 BACKGROUND

HF is a progressive and debilitating disease, the most common cardiovascular disorder, a major health problem and an epidemic disorder in the United States. The disease affects about 26 million people worldwide and causes more than 1 million hospitalizations in the United States and Europe annually.^1^ HF is one of the major causes of hospitalization in adults and the elderly, which is associated with increased morbidity and mortality and imposes a significant burden on the healthcare system.^2^ Heart disease (67.9%) including HF has been the leading cause of sudden cardiac death (SCD) as well as a major health challenge.^3^ Studies show sudden death in 25%–30% of HF patients with reduced cardiac output.^4^ HF has a significant impact on all aspects of quality of life (QoL), including physical function, mental health and social domains. According to the New York Heart Association (NYHA), the QoL disruption in patients with HF is higher than those with other cardiovascular or noncardiac conditions.^5^ Although many HF treatments have a positive impact on the QoL of patients, many limitations are still available in the QoL of HF patients.^6^ These limitations are higher in HF patients with lower cardiac output.^7^ More than 8.5 million Americans suffering HF experience depressive symptoms and poor QoL.^8^

Chronic HF requiring self‐care and symptom management caused 70%–80% of healthcare costs in Europe in 2013.^9^ Failure in self‐care leads to the use of health systems and increased health care costs.^10^ Self‐care in HF is a two‐stage process: the first one involves self‐care in everyday behaviors and the second one is self‐care management, including knowing changes in behaviors and responding to them. The self‐care process is affected by self‐efficacy.^9^ Many studies are being conducted to improve drug admission, reduce forgetfulness, and activate self‐efficacy and motivation in patients. However, social support is one part of the patients' health that affects the patient's family. Social support can improve the QoL and self‐efficacy of patients.^11^ As mentioned above, the risk of SCA in HF patients is high due to dysrhythmias. Implantable cardioverter defibrillator (ICD) implantation is one of the methods for SCD prevention and survival in HF patients.^12^ ICD prevents cardiac arrest by evacuating electrical shock. ICD is more effective than medication for ending cardiac dysrhythmia.^13^ In primary and secondary preventions, ICD is fundamental for preventing SCD.^14^, ^15^

The American Heart Association (AHA), the American College of Cardiology (ACC), and the Heart Rhythm Society (HRS) have recommended ICD for the primary prevention of SCD in patients with known criteria.^16^ Previous studies have shown that the refusal to use ICD for primary prevention is common due to patients' negligence, low risk of SCD, and the lack of medical advice.^17^ Although many guidelines have been published for embedding ICD for patients at risk, the embedding has been less done because of not being well‐perceived. Many barriers are available to implant ICD including no recognition of ICD implications, the absence of a heart surgeon and necessary resources, the high cost of embedding ICD device, and doubts about the benefits of ICD. If cardiologists believe that embedding an ICD device is not very beneficial, they will not propose an ICD for the patient.^18^

HF patients do not understand the risk well. Physicians must inform patients in a meaningful way and help them understand the purpose, risks and benefits of their treatment and reach an informed choice.^18^ But using this device may be challenging for patients and their families.^19^ The ICD affects some aspects of people's lives, including social and family relationships, physical activity, psychological state, lifestyle, and QoL.^13^, ^20^, ^21^ No study in Iran was found on the primary prevention of ICD in patients at risk of SCD, especially those with HF and reduced cardiac output. Therefore, the present study was conducted with the following specific objectives. (a) the evaluation of knowledge about HF and ICD; (b) the evaluation of concerns about ICD; (c) assess the four variables of perceived symptoms, self‐efficacy, perceived social support, and QoL; (d) the correlation among ICD barriers (knowledge and concerns) and perceived symptoms, self‐efficacy, perceived social support, and QoL.

## 2 MATERIALS

### 2.1 Study design and setting

The present study had a cross‐sectional design. We evaluated some barriers of the ICD implantation for the primary prevention of SCD in HF patients in southeast Iran.

### 2.2 Sample size and sampling

A descriptive cross‐sectional study was conducted among 147 HF patients with reduced ejection fraction. The HF patients were admitted to two public‐educational hospitals in different wards cardiac care unit (CCU) in Ali Ibn Abitaleb hospital of Rafsanjan city and shafa hospital of Kerman city. They were discharged after receiving the necessary care in the critical care unit (CCU) in one of the southeastern cities of Iran. The inclusion criteria were patients aged ≥21 years old, diagnosed with HF and reduced cardiac output (Left ventricular ejection fraction <35%) based on NYHA criteria, the presence of a formal or informal caregiver, and signed written informed consent. Patients with visual and auditory processing disorders, severe neurological or psychiatric disorders (e.g., Parkinson's disease, Alzheimer's disease), and an uncompleted questionnaire were excluded from the study.

### 2.3 Data collection

Two hundred questionnaires were distributed in the two hospitals over a 14‐month period (From April 2018 to June 2019) and 170 copies were returned (response rate: 85%). After poorly completed questionnaires (e.g., they were not fully completed) were excluded, data from 147 participants were used in the final analyses. Thus, the sample had an appropriate size.

### 2.4 Measurements

Four questionnaires were used to collect data. The first one consisted of demographic and disease profile information (gender, marital status, income, educational level, employment status, duration of heart failure, history of hospitalization, number of admissions, ejection fraction, functional class, and other illnesses).

The second questionnaire was a researcher‐made one by previous studies which examined: (a) the severe symptoms of HF in a 19‐item table based on a five‐point Likert scale (never = 0, rarely = 1, sometimes = 2, often = 3, and always = 4) with total score of 0–86, (b) patients' worries about using ICD in a 16‐item table based on a five‐point Likert scale (totally disagree, disagree, no idea, agree, totally agree). The scores range between 0 and 26 points; (c) HF patients' knowledge of the consequences or outcomes of heart failure and ICD (4 items with five responses in which only one answer is correct); with the total score of 0–4. (d) Seven items about knowledge of device therapy and decisions for ICD implantation; two items for being informed about ICD and the physician's advice for ICD implantation with yes or no answers; one item about “from whom did you hear about ICD”; four other questions studied the patient's viewpoints about ICD implantation. The validity of the questionnaire was assessed using content validity, and reliability was assessed using internal consistency. Cronbach's *α* coefficient was 0.96.

The general self‐efficacy beliefs scale (GSE‐10) is a 4‐point one (1: *not at all true*; 2: *hardly true*; 3: *moderately true*; and 4: *exactly true*) with the total score of (10–40) used to predict adaptability after the transformations. High reliability, stability, and construct validity of the GSE‐10 have confirmed in the study.^22^ The Cronbach *α* coefficient of the GSE‐10 scale in Iran was 0.844, which was standardized by Rajabi.^23^ In the present study, the Cronbach's *α* for the GSE‐10 scale was 0.94.

World Health Organization Quality of life‐BRE (WHOQOL‐BREF) with 26 items includes psychophysical health, social relationships, and environment as a QoL to measure health. Two items of health and QoL are unscored. The score for each item ranges from 1 to 5 (never, low, medium, high, and quite a lot). Internal consistency of the study was excellent (0.92) and test–retest reliability was good.^24^ The Cronbach's *α* coefficient of WHOQOL‐BREF in Iran was 0.78.^25^ The short version should be converted to the long version, and then the QoL was interpreted from zero to 100. In the present study, Cronbach's *α* for the WHOQOL‐BREF scale was 0.89.

Multidimensional Scale of Perceived Social Support (MSPSS) contains 12 items based on seven‐point Likert scale (very strongly disagree to very strongly agree). The MSPSS individually measures the PSS through three sources: significant others, family and friends. The Cronbach's *α* coefficient of MSPSS in Iran was 0.93.^26^ The score ranges from 12 to 72 points. In the present study, Cronbach's *α* for the MSPSS scale was 0.95.

### 2.5 Data analysis

Data were analyzed using SPSS22 and descriptive and inferential statistical methods. Descriptive statistics, including frequency, percentage, mean and standard deviation were used to describe demographic characteristics and mean scores (knowledge about HF and ICD; concerns about ICD). Mean and standard deviation were used to describe self‐efficacy, QoL, PSS. Analysis of variance and independent *t*‐test, and in some cases Mann–Whitney *U* and Kruskal–Wallis tests were used to determine the relationship between demographic and disease profile information questionnaire, knowledge about HF and ICD, and concerns about ICD. Spearman correlation coefficient was used to determine the relationship between Knowledge about HF and ICD, Concerns about ICD, self‐efficacy, QoL and PSS. Also, *p *value and 95% confidence intervals are reported. A significance level of 0.05 was considered.

### 2.6 Ethical considerations

This study was conducted after obtaining the ethics license (IR.RUMS.REC.1396.112) from the Ethics Committee of Rafsanjan University of Medical Sciences. Before sampling, informed written consent was taken from HF patients, who were explained about the objectives of the study, confidentiality and anonymity of the information and the voluntary participation in the study and voluntary withdrawal at any time. They were explained that participating in or withdrawing from the study would not affect their course of treatment, and all their information would remain confidential.

## 3 RESULTS

### 3.1 Respondent demographics

The mean age of the participants was 67.68 ± 14.37 years. The mean score of ejection fraction was 22.38 ± 7.59. The majority of the participants were male (56.5%; *n* = 83), married (88.4%; *n* = 130), illiterate (54.1%; *n* = 79), and unemployed (44.5%; *n* = 65). The majority of HF patients have a history of hospitalization (95.2%; *n* = 140) (Table 1). The results also showed a positive correlation among the knowledge about HF, ICD score, and the duration of HF (*p* < 0.05). In addition, the knowledge about HF and ICD scores of married participants were more than that of the widowers. The participants with more than monthly two million‐toman income had much knowledge compared to others. Also, either retired or employed participants had much knowledge compared to self‐employed or unemployed ones. Among the demographic variables, only age was significantly correlated with the concerns about ICD score (*p* < 0.05) (Table 1).

**Table 1:** Demographic and clinical information of the participants (*n* = 147).

Variables	Mean (SD)	Knowledge about HF and ICD		Concerns about ICD	
		Spearman correlation coefficient	*p* value	Spearman correlation coefficient	*p* value
Age (year)	67.68 (14.37)	0.01	0.91	0.18	*p* < 0.05
Duration of HF (year)	5.32 (4.88)	0.27	0.001	0.09	*p* > 0.05
Ejection fraction (%)	22.45 (7.66)	0.01	0.90	0.04	*p* > 0.05

	*N*(%)	Statistical test	*p* value	Statistical test	*p* value
Gender					
Male	83 (56.5)	*Z* = −1.87	0.06	*Z* = −1.95	*p* > 0.05
Female	64 (43.5)				
Marital status					
Married	130 (88.4)	*Z* = −2.84	0.005	*Z* = −0.46	*p* > 0.05
Widowed	17 (11.6)				
Income (million riyal)					
<0.5	36 (25.0)	*H* = 19.32	<0.001	*H* = 2.24	*p* > 0.05
0.5−1	35 (24.3)				
1–2	59 (41.0)				
<2	14 (9.7)				
Educational level					
Illiterate	79 (54.1)	*H* = 6.68	0.08	*H* = 0.36	*p* > 0.05
<Diploma	51 (34.7)				
Diploma	9 (6.1)				
Academic	7 (4.8)				
Employment status					
Employed	6 (4.1)	*H* = 12.30	0.006	*H* = 3.11	*p* > 0.05
Self‐employed	41 (28.1)				
Retired	34 (23.3)				
Unemployed	65 (44.5)				
History of hospitalization					
Yes	140 (95.2)	*Z* = −1.32	0.19	*Z* = −0.35	*p* > 0.05
No	7 (4.8)				
Other Illnesses					
Yes	99 (67.3)	*Z* = −0.54	0.59	*Z* = −0.51	*p* > 0.05
No	45 (30.6)				

*Note*: Data were presented as number (%). The sample consisted of 147 HF patients with mean age 67.68± 14.37 years.

Abbreviations: HF, heart failure; ICD, implantable cardioverter defibrillator; *H*, Kruskal–Wallis test; SD; standard deviation; *Z*, Mann Whitney *U* test.

62.6% (*n* = 92 of the 147 patients) of the participants did not know about HF and ICD and the rest (37.4%, *n* = 55 of the 147 patients) only had knowledge about some of the questions. No body answered the questions about HF and ICD completely correctly (Table 2).

**Table 2:** Participants’ knowledge about HF and ICD (*n* = 147).

Knowledge questions	True (%)	95% confidence interval
The most common result of HF	7 (4.8)	1.4–8.8
What can be done to prevent sudden death in patients with severe HF	6 (4.1)	1.4–7.5
The most common symptom of HF	51 (34.7)	27.2–42.2
The most important effect of ICD	6 (4.1)	1.4–7.5

*Note*: Data were presented as number (%).

Abbreviations: HF, heart failure; ICD, implantable cardioverter defibrillator.

The total score of patients' concerns about using ICD was 47.11 ± 11.26, which showed a moderate level (the midpoint of the questionnaire was 48). The participants were mostly worried about high surgical cost, ICD malfunction, side effects of the ICD, limited use of microwave, being old, and being dependent on others (Table 3).

**Table 3:** The participants’ concerns about using ICD (*n* = 147).

Items	Strongly agree	Mean	SD
Fear of surgery	46 (31.5)	2.97	1.28
High surgical cost	49 (34.3)	3.12	1.26
Being old	51 (35.2)	3.03	1.3
Side effects of the ICD	55 (37.9)	3.07	1.22
Not having information about ICD	45 (31.0)	2.98	1.20
Requiring special care after inserting the ICD	50 (34.7)	2.98	1.25
Fear of being dependent on others	42 (29.0)	3.01	1.16
The ICD malfunction	54 (36.7)	3.12	1.27
Use For lifetime	52 (35.9)	3.0	1.28
Restriction for swimming	38 (26.2)	2.83	1.18
Restriction for traveling by airplanes	43 (29.7)	2.99	1.10
Restriction for using microwave	42 (28.8)	3.06	1.15
Restriction for using some diagnostic tests such as MRI	40 (27.2)	2.83	1.24
Restriction for lifting heavy things	47 (32.0)	2.85	1.33
Restriction for using electrical appliances	44 (30.1)	2.96	1.18
Restriction for sexual activity	36 (24.5)	2.81	1.18
Total	‐	47.11	11.26

Abbreviations: ICD, implantable cardioverter defibrillator; MRI, magnetic resonance imaging; SD, standard deviation.

The results showed that 52.3% (*n *= 77 of the 147 patients) of the participants, decided themselves about the ICD implantation, in particular, they decided not to proceed with an ICD implantation. 82.9% (*n* = 122 of the 147 patients) of the participants announced no physician's advice or recommendation for ICD implantation. However, the participants obtained their information about ICD mostly from their physicians (12.9%; *n*  = 19 of the 147 patients) and friends (16.3%; *n*  = 24 of the 147 patients), respectively. Also, the patients' responses to the question “if the doctor recommended you again for ICD implantation, would you accept it?” were 23.2% (definitely yes), 29.9% (probably yes), 12.2% (probably no), 25.2% (definitely no), and 9.5% (unsure). The total score of perceived symptoms of HF was 38.71 ± 14.26, which showed a moderate level. The most common symptoms were dyspnea in daily activity (2.97 ± 1.15), decreased libido (2.96 ± 1.29), fatigue (2.89 ± 1.14), disability in physical activity (2.88 ± 1.17), respectively, and the least common symptom was weight gain (0.64 ± 1.19) (Table 4).

**Table 4:** Common perceived symptoms of HF among the participants (*n* = 147).

Symptoms of the disease	Most of the time	Mean	SD
Cough (except cases of colds)	51 (35.2)	1.88	1.41
Wheeze	66 (44.9)	2.0	1.43
Orthopnea	68 (46.3)	2.27	1.44
Dyspnea at rest	69 (46.9)	2.25	1.39
Dyspnea during daily activity	101 (68.7)	2.97	1.15
Disability in physical activity	97 (67.4)	2.88	1.17
Fatigue	102 (69.4)	2.89	1.14
General weakness	66 (45.8)	2.26	1.33
Confusion	42 (28.8)	1.62	1.30
Dizziness	50 (34.2)	1.72	1.43
Depression	55 (37.4)	1.83	1.50
Palpitation	53 (36.3)	1.89	1.46
Chest pain	52 (35.4)	1.85	1.37
Loss of appetite	74 (50.3)	2.17	1.47
Weight loss	45 (30.6)	1.38	1.47
Weight gain	15 (10.2)	0.64	1.19
Swelling in the ankles	59 (40.1)	1.84	1.54
Flatulence	58 (39.5)	1.87	1.39
Decreased libido	104 (77.0)	2.96	1.29
Total	‐	38.71	14.26

*Note*: Data were presented as number (%).

Abbreviations: HF, heart failure; SD, standard deviation.

The mean total score of self‐efficacy was 21.39 ± 7.05, which was lower than 25 (cutoff point = 25). Therefore, the self‐efficacy of the participants was less than moderate. The mean total score of perceived social support was 4.08 ± 1.44, which was higher than the median score of the questionnaire (score = 3.5). The mean total score of QOL was 40.72 ± 12.47, which was lower than the median score of the questionnaire (score = 50.0). Therefore, the QOL of the participants was less than moderate (Table 5).

**Table 5:** Self‐efficacy, PSS scores, and QOL among the participants (*n* = 147).

Variables	Mean	SD	Min	Max
Self‐efficacy	21.39	7.05	10	40
PSS	4.08	1.44	1	7
QoL	40.72	12.47	17.31	89.0

Abbreviations: QoL, quality of life; PSS, perceived social support; SD, standard deviation.

The score of knowledge about HF and ICD had a significant positive poor correlation with self‐efficacy, perceived social support, and QoL (*p* < 0.05). Also, the score of concerns about ICD had a significant negative poor correlation with perceived social support (*p* < 0.05) (Table 6). We performed a follow up of the participants' outcome 10 months after inclusion, that is, from December 1 to 11 2019 by calling the participants. Of 147 participants, 53.7% (*n* = 79) were alive, 32.7% (*n* = 48) had passed away, and 13.6% (*n* = 20) did not answer to the telephone contacts. Forty‐two cases of death were due to SCD and six cases to other reasons. Only four participants had been implanted with an ICD. Forty‐two cases of death were due to SCD and six cases for other reasons. Only four participants had been implemented ICD.

**Table 6:** The correlation among ICD barriers (knowledge and concerns) and perceived symptoms, self‐efficacy, PSS, and quality of life.

Variable	Knowledge about HF and ICD		Concerns about ICD	
	Spearman correlation coefficient	*p* value	Spearman correlation coefficient	*p* value
Perceived symptoms	−0.11	0.19	0.06	*p* > 0.05
Self‐efficacy	0.17	0.04	−0.12	*p* < 0.05
PSS	0.22	0.006	−0.18	*p* < 0.05
Quality of life	0.19	0.02	−0.15	*p* < 0.05

Abbreviations: HF, heart failure; ICD, implantable cardioverter defibrillator; PSS, perceived social support; SD, standard deviation.

## 4 DISCUSSION

Avoiding ICD implantation is common among patients who are candidates for the SCD primary prevention.^17^ The current study aimed to study barriers to the ICD implantation among HF patients with reduced ejection fraction. 62.6% of HF patients in the current study did not know about ICD. Like in our study, Yuhas et al. demonstrated that patients had a poor knowledge concerning the risk of SCD.^17^ Chan et al., also signed out patients' poor knowledge about the role of ICD in the primary prevention of SCD: about 68% of the patients believed that medical treatment could prevent SCD. Such poor knowledge was the most important factor affecting patients' ICD acceptance in that study.^27^ Lewis et al. reviewed 25 studies on patients' decisions about the ICD. They found that most patients did not understand the ICD function well and overestimated its benefits.^28^ Ottenberg et al. showed patients' poor knowledge about the goal and function of the cardiovascular implantable electronic device (CIED) and they suggested targeted training: patients should be advised to improve their learning and transfer their knowledge.^29^ Since most patients who were candidates for the ICD implantation were asymptomatic, physicians should review and discuss treatment choices for patients to reduce their concerns

The total score of patients' concerns about using the ICD in the present study was moderate. Most patients were worried about high surgical cost, the ICD malfunction, side effects of the ICD, limited use of microwave, oldness, and dependency on others. Unlike the current study, the patients' concerns in the study of Matlock et al. were about the ICD futility.^30^ According to Yuhas et al., recall, malfunction and surgical risks of the ICD were common patients' concerns.^17^ Chan et al. showed that participants were mostly concerned about restrictions in their current lifestyles including the inability to lift heavy objects, problems when working with electric appliances or traveling by airplane, and inability in swimming or sexual activity.^27^ patients' concerns are different depending on their cultural contexts. The study of Ottenberg et al. showed reduced sense of wellbeing, happiness, acceptance in future, negative experiences with the CIED and not accepting the risks of CIED implantation.^29^ Such results suggest patients' poor perception of the risks and advantages of the ICD and their improper anticipations. Despite various concerns in patients, Groarke et al. showed that 93% of the patients were satisfied with the ICD implantation.^31^ The results suggest that patients are worried about the ICD implantation because they have poor knowledge and perception. When patients accept the ICD implantation, they will be satisfied with their correct decision.

However, physicians play an important and effective role. Matlock et al. showed in their study that patients followed their physicians’ advice regardless of the risks and advantages of the device.^30^ 53.1% of the patients in the current study were satisfied with the ICD implantation in case of the physician's advice suggesting the effect of physicians on patients' decisions about the ICD implantation. According to Chan et al., 98% of the patients trusted their physicians’ information about the ICD.^27^

Physicians did not recommend the ICD implantation for 82.9% of the patients in the current study. According to one national study on 3000 doctors selected from the American medical association, doctors’ beliefs are an important barrier to ICD implantation and SCD prevention.^32^ According to Yuhas et al., patients did not accept the ICD implantation because doctors did not advise them to do so. Also, many patients had a poor perception of the risks of ICD and lifestyle changes. This poor perception was attributed to their doctors.^17^ Lewis et al. found that doctors' advice was effective in improving patients' knowledge and decision about the ICD implantation; since the ICD implantation is related to complicated issues of life and mortality, it is difficult for patients to decide about it.^28^ It is not surprising that patients trust their physicians because they are specialists. Doctors can support patients better by reviewing patients' understanding and advantages and they can provide better treatment outcomes for patients through joint decisions. Carroll et al. found that doctors’ advice and information about SCD were factors affecting the ICD acceptance. The decision‐making process is mostly affected by patients' trust, social effects, and health.^33^

According to the current study, knowledge about the HF and ICD had a significant poor relationship with self‐efficacy, perceived social support, and QoL showing the poor effect of such factors on the ICD acceptance. Although the effects of the above factors on the ICD acceptance have not been mentioned in other studies, HF patients have poor QoL and social support^34^, ^35^ leading to an increased risk of hospitalization and mortality. Also, HF patients may not accept their illnesses due to their poor QoL and thus they do not participate in the treatment process.^36^ HF patients' self‐efficacy is effective on their self‐care and illness management.^37^ The relationship between knowledge and self‐efficacy can affect patients' decisions about the ICD implantation.

According to the current study, concerns about the ICD had a poor significant correlation with perceived social support and QoL. Although the effects of the above factors on the ICD acceptance have not been mentioned in other studies, Bosworth et al. found that HF patients were mostly worried about the illness prognosis uncertainty and cognitive function^38^ which can affect the perception of illness symptoms and control as well as the ICD acceptance. Since perceived social support has been significantly correlated with the QoL^39^ in many studies, these two factors can affect patients' concerns and decisions. Therefore, doctors should understand a wide range of concerns in HF patients.

Although 52.3% of the participants in the current study decided about the ICD implantation themselves, a result which is close to that of Chan et al. (61%), many other factors are available about patients such as patients' disagreement with physician's advice,^27^ patients' feelings about long‐term interventions and their power in understanding physicians’ advice.^17^ Chan et al. showed that patients' perceptions of the physician's advice were an important predictor of the ICD acceptance and only 8% of the patients perceived the role of ICD in preventing the SCD.^27^ Therefore, healthcare providers should perceive the risk of SCD in such patients and identify factors affecting patients' decisions.^33^ However, according to Bernier et al., 52% of patients who avoided the ICD implantation, had no logical reasons. The most common reason was patients' preference (48%) for no ICD implantation. The predictors related to the ICD rejection were ages above 75 years old and a history of cancer.^40^ The age in the current study was correlated with concerns about the ICD implantation as well. Also, the current study showed that knowledge of HF and ICD was significantly correlated with HF duration, marital status, monthly incomes over two million tomans, being either retired or employed. The study of Chan et al. did not show such a significant relationship^27^ because they did not study the correlation between patients' knowledge and these variables. Studies suggest that a small number of patients tried to acquire knowledge about ICD. Most participants in the current study (16.3%) obtained their information from their friends. Unlike the current study, Chan et al. showed that most participants (16.3%) obtained their information from publications.^27^ Such a difference may be due to the patients' poor level of education in the current study.

### 4.1 Limitations

The main limitation of this study was the very low educational level of the majority of the participants; educated patients may be more aware of HF as well as the ICD indication and procedure. Also, we did not interview the physicians to know doctor‐related misperceptions and recommendations. In addition, this study had a cross‐sectional design so that we could not interview patients who accepted the ICD implantation and had valid knowledge and recognition regarding the ICD procedure and the reasons for using it.

## 5 CONCLUSION

This study helps increasing knowledge about the barriers concerning ICD implantation in HF patients with an indication for primary prevention of SCD. Results suggest that poor knowledge about ICD, patients' concerns about ICD, and no physician's advice are the most important reasons for rejecting the device. However, the perceived social support, QoL, and educational level seem to be indirect poor factors affecting the patients' acceptance and they are also effective on the knowledge about HF, ICD necessity as well as concerns about the ICD. Physicians should take measures to train and educate patients. Further research on factors affecting the ICD implantation for primary prevention should be done in different populations.

### 5.1 Relevance to clinical practice

It is important to assess what patients think and concern about the risks of not inserting ICD, how much they are aware of these risks, and how important these therapeutic recommendations are for them. It is helpful to investigate and remove the concerns and factors influencing the insertion of an ICD in patients. These results can help healthcare professionals choose the correct device as well as make the right decision when training the patients, and thus the chance of survival in patients increases.

## AUTHOR CONTRIBUTIONS

**Mohammad A. Zakeri**: Conceptualization; investigation; supervision; validation; writing—original draft; writing—review and editing. **Nadia Sedri**: Conceptualization; data curation. **Golamreza Bazmandegan**: Conceptualization; supervision. **Maryam Zakeri**: Conceptualization; data curation; investigation. **Mohammad Safariyan**: Conceptualization; investigation. **Mahlagha Dehghan**: Conceptualization; formal analysis; investigation; supervision; validation; writing—review and editing. Mahlagha Dehghan had full access to all of the data in this study and takes complete responsibility for the integrity of the data and the accuracy of the data analysis.

## CONFLICT OF INTEREST

The authors declare no conflict of interest.

## ETHICS STATEMENT

The code of ethics (IR.RUMS.REC.1396.112) was received from the Ethics Committee of Rafsanjan University of Medical Sciences.

## TRANSPARENCY STATEMENT

The Mahlagha Dehghan affirms that this manuscript is an honest, accurate, and transparent account of the study being reported; that no important aspects of the study have been omitted; and that any discrepancies from the study as planned (and, if relevant, registered) have been explained.

## Data Availability

The data used to support the findings of this study are available from the corresponding author upon request.
